# Supplementary material for: Association of body mass index with health care expenditures in the United States by age and sex
Source: PLoS One. 2021 Mar 24;16(3):e0247307. doi: 10.1371/journal.pone.0247307 (PMC7990296; doi:10.1371/journal.pone.0247307)
Supplement: S1 File — Additional methodological details and results. (PDF) [file pone.0247307.s001.pdf]

Supplemental Appendix to: Association of body mass index with health care expenditures in the US by age and sex

Zachary J. Ward, Sara N. Bleich, Michael W. Long, Steven L. Gortmaker

Contents

1 Datasets **2**
1.1 Price Adjustment 2
1.2 Exclusion Criteria 2
1.3 Variable Coding and Standardization 3
1.4 Respondent Characteristics 3

2 Adjustment For Self-Report Bias **5**

3 Expenditure Standardization **12**
3.1 Part I: Probability of Positive Expenditure 13
3.2 Part II: Level of Expenditure 16
3.3 Full Model Standardization 19

4 BMI-related Expenditure Prediction **20**
4.1 Continuous 20
4.2 Categorical 21

5 Model Uncertainty **22**

6 Comparisons to Previous Estimates **23**
6.1 Finkelstein 2008 23
6.2 Wang 2015 25
6.3 Cawley and Meyerhoefer 2012 26

1 Datasets

We used data from the Medical Expenditure Panel Survey (MEPS) 2011-2016. The following Full Year Consolidated Data Files were used:

Year	File
2011	HC-147
2012	HC-155
2013	HC-163
2014	HC-171
2015	HC-181
2016	HC-192

Variable definitions for Race and Education were harmonized across survey years as needed. Unfortuantely, adult BMI was not available in the 2017 MEPS data, so we could not use the latest round of data in this analysis.

1.1 Price Adjustment

Following the MEPS guidelines for pooling total expenditures (https://meps.ahrq.gov/about_meps/Price_Index.shtml) we adjusted total expenditures using the index of Personal Consumption Expenditures - Health (PCE-Health) to standardize expenditures in $US 2019.

Year	PCE-Health
2011	98.029
2012	100
2013	101.228
2014	102.635
2015	103.772
2016	105.449
2017	107.250
2018	109.127
2019	110.675

Source: Bureau of Economic Analysis (https://www.bea.gov/) [accessed 08/24/2020] National Income and Product Accounts Section 2 Personal Income and Outlays Table 2.5.4 Price Indexes for Personal Consumption Expenditures by Function (A) Line 37 Health

1.2 Exclusion Criteria

The pooled dataset contained 150,043 adults (20+) and 66,141 children (<20). We excluded women who reported being pregnant in any portion of the panel because of possible effects on weight and medical expenditures, leaving 145,067 adults and 65,797 children. We then excluded individuals with no reported BMI, leaving 139,150 adults and 36,613 children (BMI is not available in MEPS for children younger than 6). We removed children with implausible BMI, <5 (n=3) or >100 (n=7), leaving 36,603 children.

In addition, 4 adults were missing Region, 3 adults were missing Marital Status, and 20 children were missing Region. Our final dataset thus contained contained 175,726 respondents – 139,143 adults (aged 20 and older) and 36,583 children (ages 6-19).

Nearly all of the respondents (99.7% of adults and 99.8% of children) were in scope for the entire reference period of the survey, so we did not adjust for scope of the reference period.

1.3 Variable Coding and Standardization

Adults with no reported education level (Not ascertained/DK/Refused) (n=1512) were pooled into one category and used as the reference group.

Categorical variables were replaced by dummy variables (0/1), and continuous variables were modeled as cubic polynomials for greater flexibility. Continuous variables were standardized to all have mean 0 and variance 1, and higher order terms (squared, cubed) were re-calculated from the standardized base.

1.4 Respondent Characteristics

Here we report the sample-weighted means/percentages of respondent characteristics among our sample of 175,726 respondents.

Variable	Children: Mean (SD)	Adults: Mean (SD)
Total Expenditure	2101.03 (7804.19)	5813.23 (16640.59)
BMI (self-reported)	21.27 (5.57)	28.04 (6.43)
Year	2013.5 (1.71)	2013.54 (1.71)
Age	13.12 (3.91)	48.65 (17.42)
Male (%)	51.08	49.84
Smoker (%)	1.19	15.1
Poverty Level	335.73 (283.1)	420.07 (352.58)
Region (%)		
Northeast	17.28	18.09
Midwest	21.54	21.28
South	38.08	37.34
West	23.11	23.29
Race/Ethnicity (%)		
White	74.6	79.58
Black	14.23	11.82
American Indian/Alaska Native	0.88	0.72
Asian/Native Hawaiian/Pacific Islander	5.08	5.9
Multiple Races	5.2	1.97
Hispanic	21.11	14.74
Marital Status (%)		
Married	NA	54.18
Widowed	NA	6.42
Divorced	NA	12.07
Separated	NA	2.2
Never Married	NA	25.13
Education (%)		
Less than High School	NA	4.36
Some High School	NA	7.07
GED or High School Diploma	NA	28.83
Some College	NA	28.83
College Graduate	NA	19.14
Graduate School	NA	11.23
Unknown	NA	0.54
Health Insurance (%)		
Private	61.5	66.94
TRICARE	2.04	3.17
Medicare	0.15	22.95
Medicaid	36.75	11.77
Public A	0.29	0.36
Public B	0.55	1.1

Variable	Children: Mean (SD)	Adults: Mean (SD)
None	5.22	12.48

2 Adjustment For Self-Report Bias

It is well-documented that self-reported BMI tends to underestimate adult obesity prevalence, while it may overestimate obesity prevalence among younger children. Thus, reported BMI in MEPS needs to be adjusted for self-report bias. Previous approaches to correct for self-report bias have used regression-based approaches to adjust mean BMI at the individual-level (Cawley 2006) or aggregate-level (Dwyer-Lindgren 2013). However, aligning the mean of self-reported BMI with measured data does not imply that the entire BMI distribution has been well-adjusted. Indeed, regression-based approaches reduce the variance of adjusted BMI distributions, and thus tend to underestimate obesity prevalence due to smaller tails of the predicted distribution (Ward 2016).

Using the bias-correction approach described in Ward 2019, we adjusted the entire distribution of self-reported BMI in MEPS to be consistent with the distribution of measured BMI in NHANES, which is the gold standard of population-representative body measures in the US. We pooled NHANES data from 2011-2016 which contained 17,048 adults age 20+ and 8,052 children ages 6-19. We removed pregnant adult women (n=192) and those without measured BMI (n=883), leaving 15,973 adults in our final dataset. No children reported being pregnant. After removing children without measured BMI (n=367) we had 7,685 children in the final dataset.

Here we compare the distribution of MEPS self-reported BMI with NHANES measured BMI by age group. We see significant under-reporting of BMI for both Males and Females, as characterized by the two-sample Kolmogorov-Smirnov D-statistic which is a metric for the distance between two distributions.

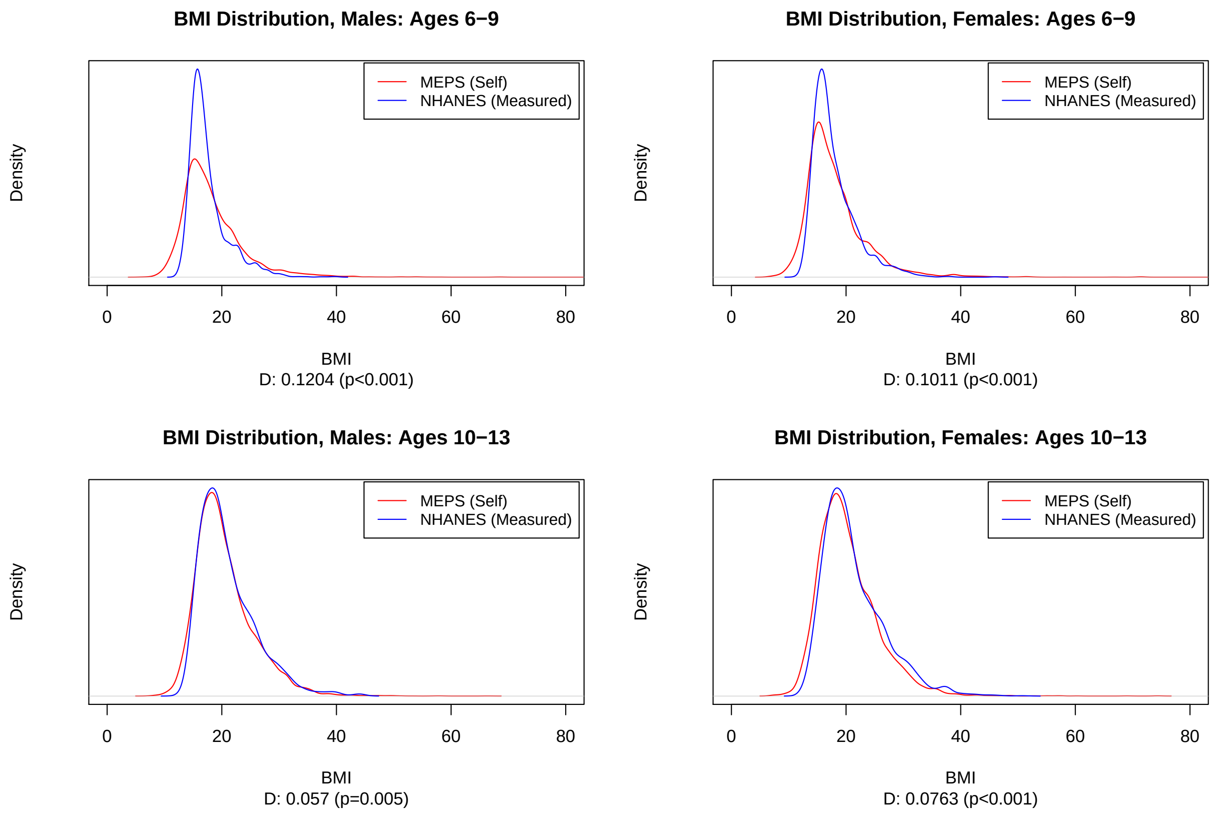

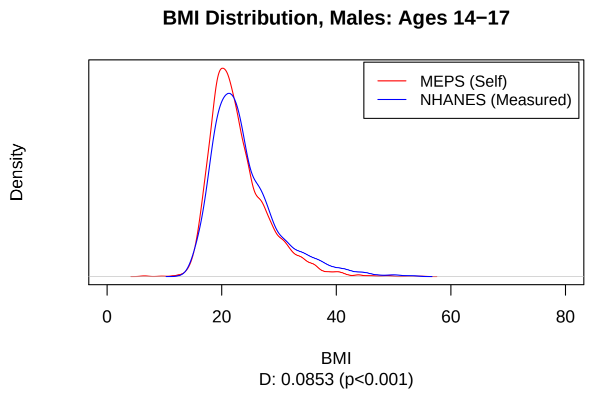
BMI Distribution, Males: Ages 14−17
MEPS (Self)
NHANES (Measured)
Density
0
20
40
60
80
BMI
D: 0.0853 (p<0.001)

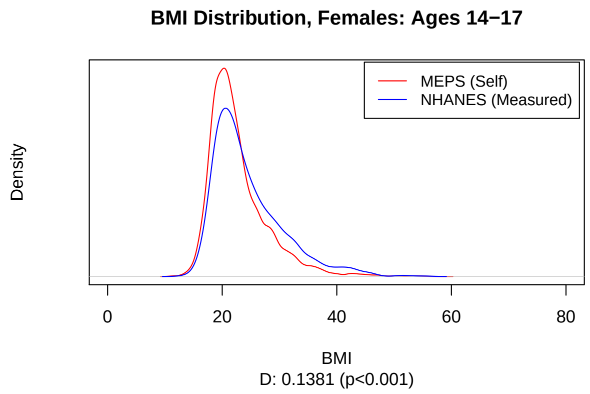
BMI Distribution, Females: Ages 14−17
MEPS (Self)
NHANES (Measured)
Density
0
20
40
60
80
BMI
D: 0.1381 (p<0.001)

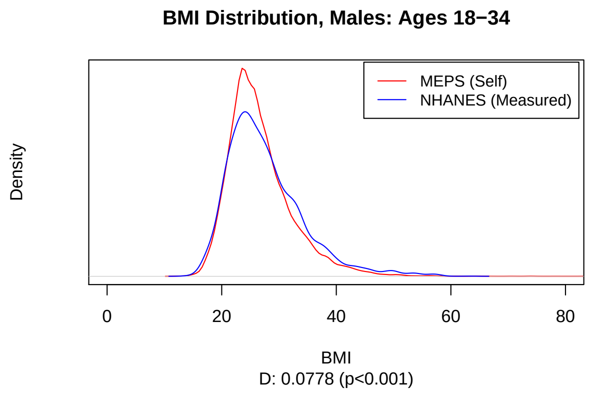
BMI Distribution, Males: Ages 18−34
MEPS (Self)
NHANES (Measured)
Density
0
20
40
60
80
BMI
D: 0.0778 (p<0.001)

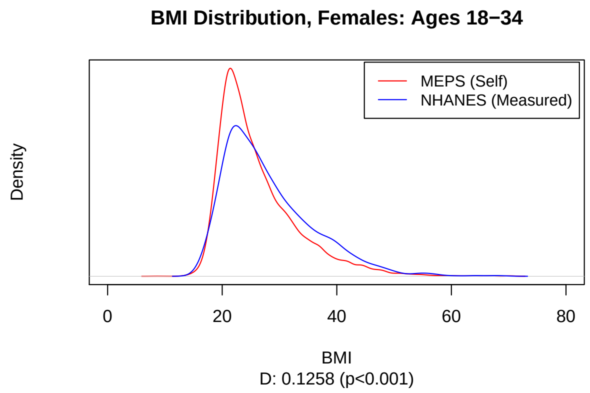
BMI Distribution, Females: Ages 18−34
MEPS (Self)
NHANES (Measured)
Density
0
20
40
60
80
BMI
D: 0.1258 (p<0.001)

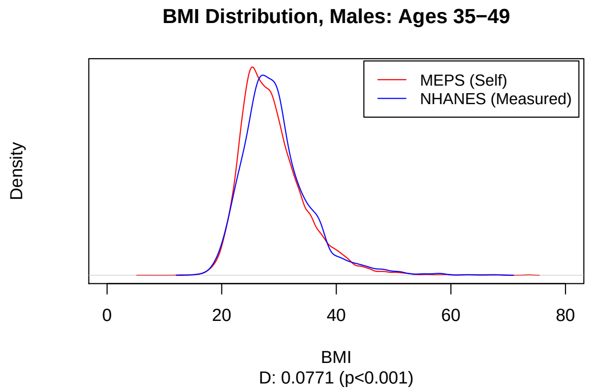
BMI Distribution, Males: Ages 35−49
MEPS (Self)
NHANES (Measured)
Density
0
20
40
60
80
BMI
D: 0.0771 (p<0.001)

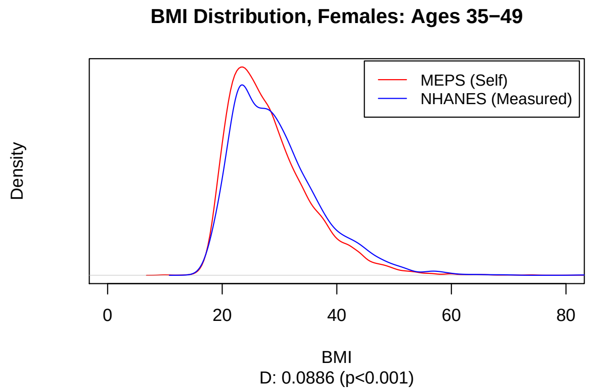
BMI Distribution, Females: Ages 35−49
MEPS (Self)
NHANES (Measured)
Density
0
20
40
60
80
BMI
D: 0.0886 (p<0.001)

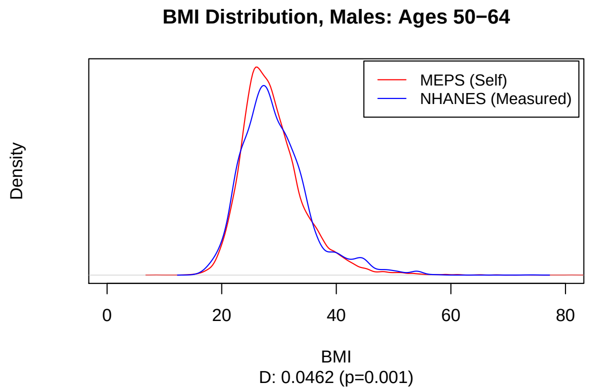
BMI Distribution, Males: Ages 50−64
MEPS (Self)
NHANES (Measured)
Density
0
20
40
60
80
BMI
D: 0.0462 (p=0.001)

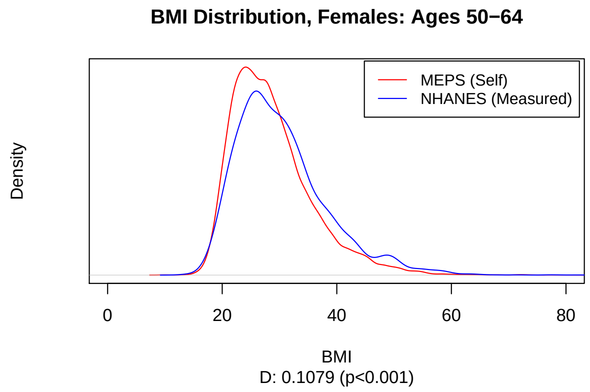
BMI Distribution, Females: Ages 50−64
MEPS (Self)
NHANES (Measured)
Density
0
20
40
60
80
BMI
D: 0.1079 (p<0.001)

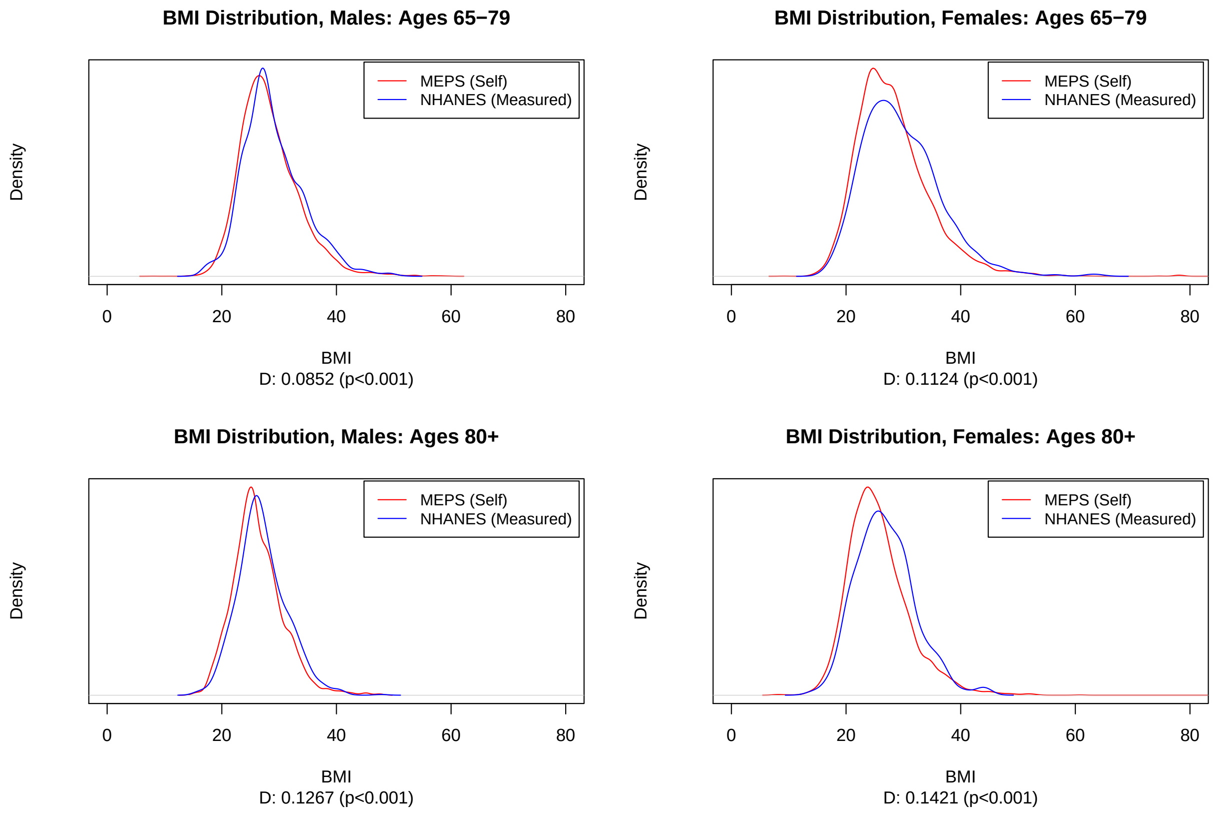

To map the distribution of self-reported BMI onto the measured NHANES distribution we estimated the (sample-weighted) quantiles (0.01-0.99) of each distribution by sex and age group. We then calculated the difference between the measured and self-reported values of BMI at each quantile and fit cubic splines with knots at each quintile to these points.

We see that self-report bias tends to increase with BMI quantile in adults.

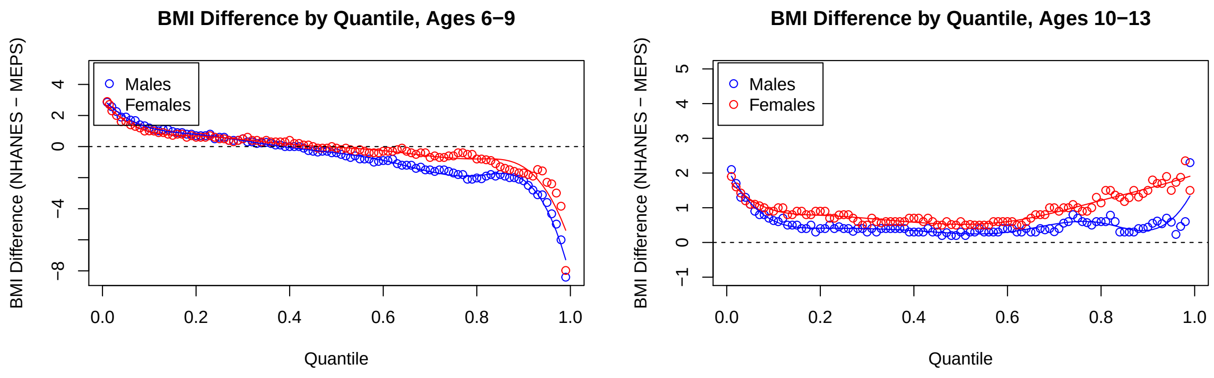

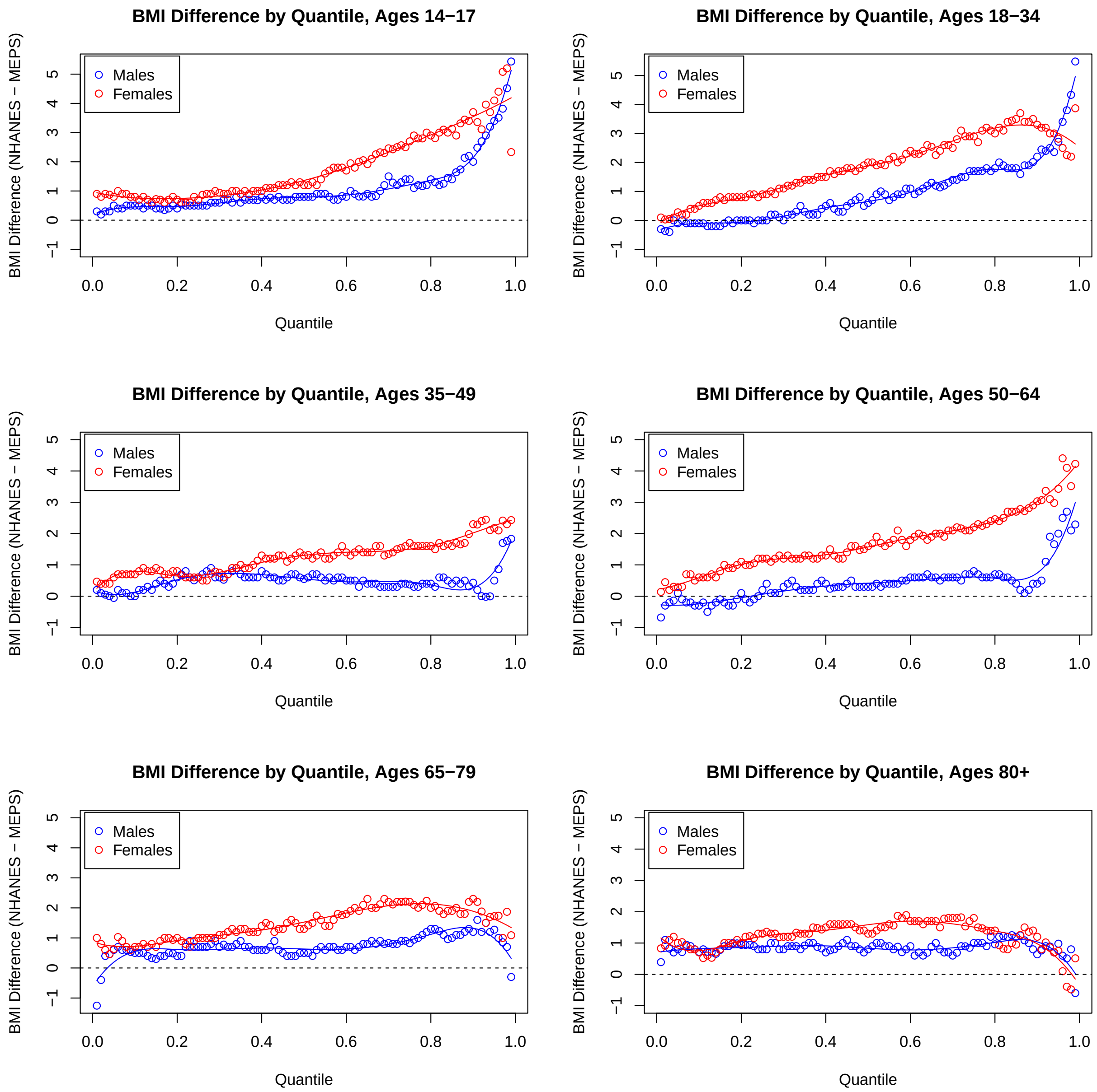

We then calculated the (sample-weighted) quantile of self-reported BMI for each respondent in MEPS and predicted their BMI difference using the fitted cubic splines. We used this predicted difference to adjust their self-reported BMI. While this approach adjusts for systematic self-report bias, it does not address rounding error that comes from reporting height/weight in discrete, rounded units. To smooth this rounding error we also perturbed BMI by adding normally-distributed noise with mean zero and standard deviation 0.5.

We see that the distributions of adjusted BMI are now statistically similar ($p>0.05$) to the measured distributions from NHANES. This also ensures that the prevalence estimates of BMI categories are similar.

BMI Distribution, Males: Ages6−9

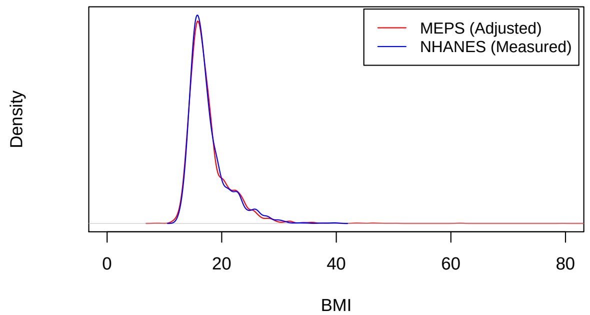

D: 0.0193 (p=0.931)

BMI Distribution, Females: Ages6−9

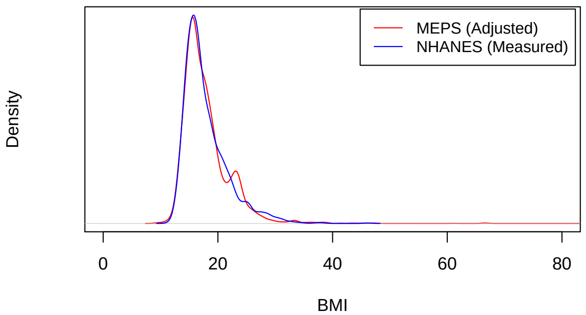

D: 0.033 (p=0.239)

BMI Distribution, Males: Ages10−13

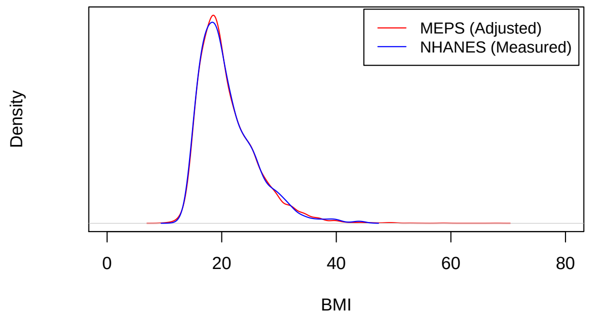

D: 0.0172 (p>0.999)

BMI Distribution, Females: Ages10−13

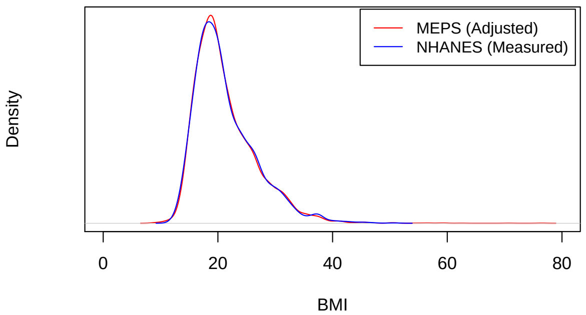

D: 0.0228 (p=0.774)

BMI Distribution, Males: Ages14−17

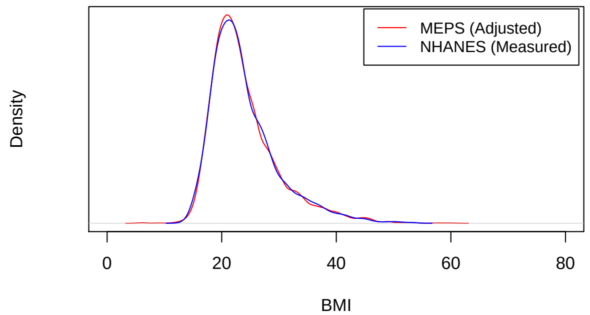

D: 0.0155 (p>0.999)

BMI Distribution, Females: Ages14−17

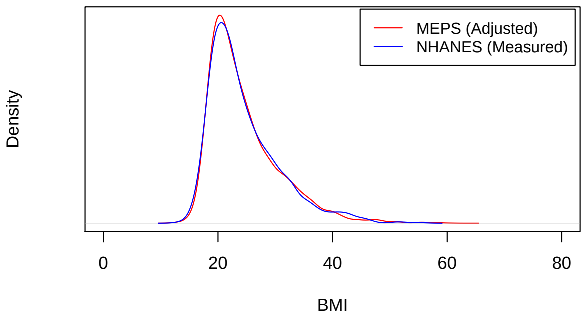

D: 0.024 (p=0.773)

BMI Distribution, Males: Ages18−34

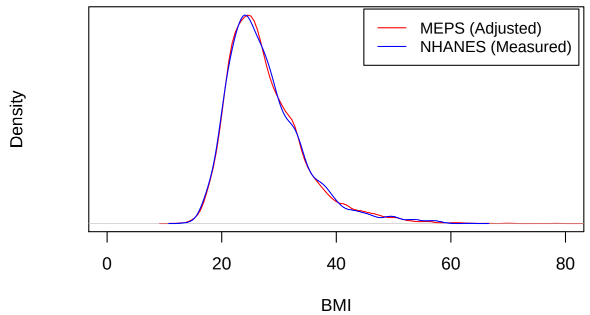

D: 0.0127 (p=0.961)

BMI Distribution, Females: Ages18−34

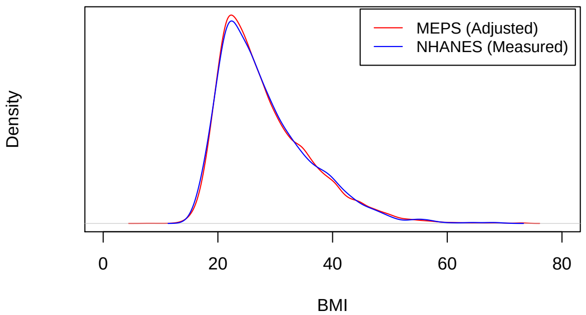

D: 0.0167 (p=0.594)

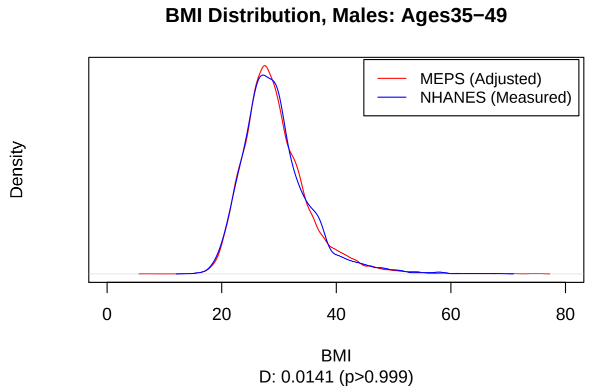
BMI Distribution, Males: Ages35−49
MEPS (Adjusted)
NHANES (Measured)
Density
0
20
40
60
80
BMI
D: 0.0141 (p>0.999)

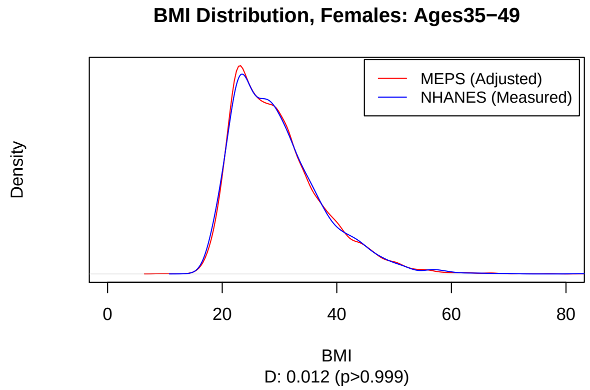
BMI Distribution, Females: Ages35−49
MEPS (Adjusted)
NHANES (Measured)
Density
0
20
40
60
80
BMI
D: 0.012 (p>0.999)

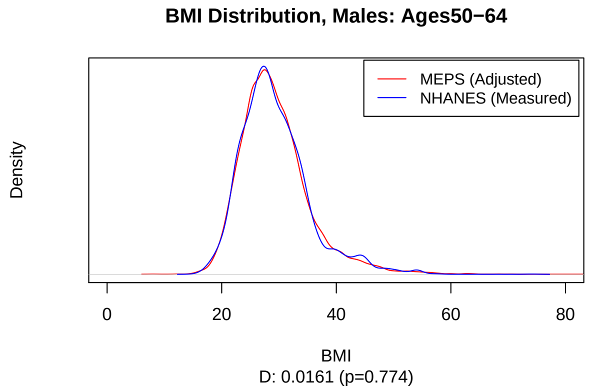
BMI Distribution, Males: Ages50−64
MEPS (Adjusted)
NHANES (Measured)
Density
0
20
40
60
80
BMI
D: 0.0161 (p=0.774)

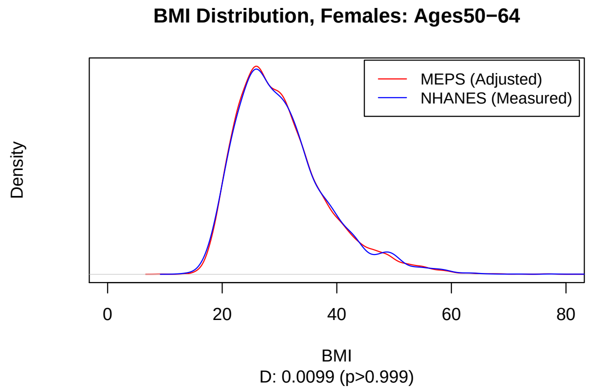
BMI Distribution, Females: Ages50−64
MEPS (Adjusted)
NHANES (Measured)
Density
0
20
40
60
80
BMI
D: 0.0099 (p>0.999)

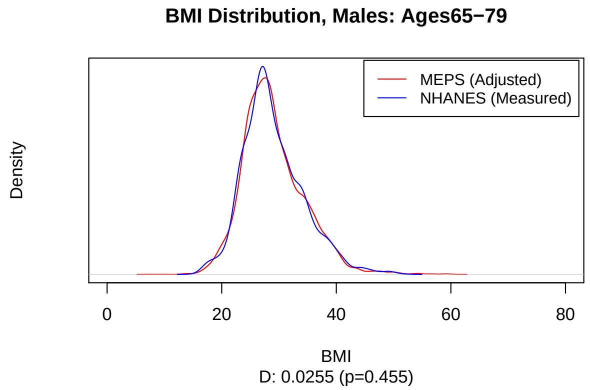
BMI Distribution, Males: Ages65−79
MEPS (Adjusted)
NHANES (Measured)
Density
0
20
40
60
80
BMI
D: 0.0255 (p=0.455)

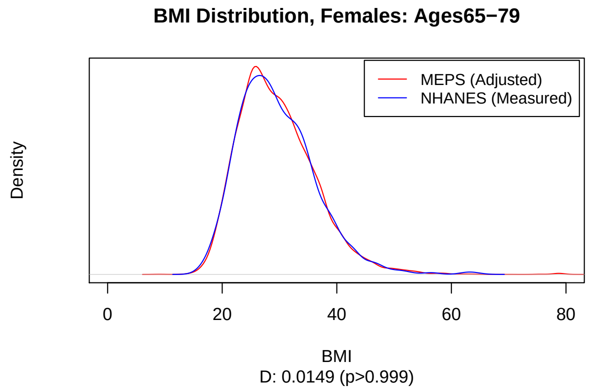
BMI Distribution, Females: Ages65−79
MEPS (Adjusted)
NHANES (Measured)
Density
0
20
40
60
80
BMI
D: 0.0149 (p>0.999)

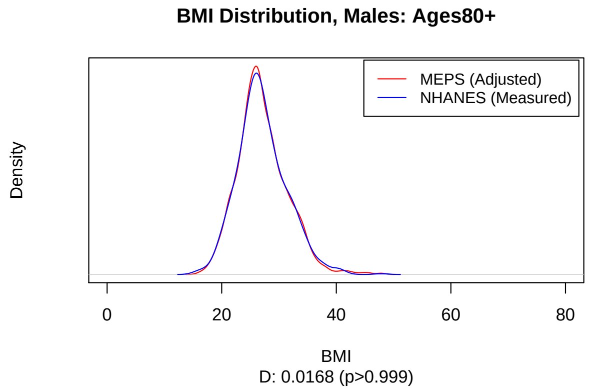
BMI Distribution, Males: Ages80+
MEPS (Adjusted)
NHANES (Measured)
Density
0
20
40
60
80
BMI
D: 0.0168 (p>0.999)

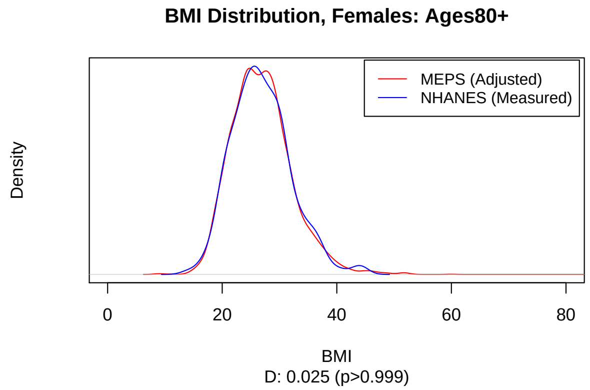
BMI Distribution, Females: Ages80+
MEPS (Adjusted)
NHANES (Measured)
Density
0
20
40
60
80
BMI
D: 0.025 (p>0.999)

References:

Cawley J, Burkhauser R. Beyond BMI: The Value of More Accurate Measures of Fatness and Obesity in Social Science Research. NBER Working Paper Series. 2006; Working Paper 12291. Available from: http://www.nber.org/papers/w12291.pdf.

Dwyer-Lindgren L, Freedman G, Engell RE, Fleming TD, Lim SS, Murray CJL, et al. Prevalence of physical activity and obesity in US counties, 2001-2011: a road map for action. Popul Health Metr. 2013;11:7.

Ward ZJ, Long MW, Resch SC, Gortmaker SL, Cradock AL, Giles C, et al. Redrawing the US Obesity Landscape: Bias-Corrected Estimates of State-Specific Adult Obesity Prevalence. PLoS One. 2016;11(3):e0150735.

Ward ZJ, Bleich SN, Cradock AL, Barrett JL, Giles CM, Flax C, et al. Projected US state-level prevalence of adult obesity and severe obesity. N Engl J Med. 2019;381(25):2440-2450.

3 Expenditure Standardization

There are factors which may influence both BMI and medical expenditures, such as smoking. Thus to estimate the relationship between BMI itself and expenditures we need to control for these other factors. Here we estimate the influence of these other factors on expenditures and create a 'standardized' population in which the effect of these identified factors on expenditures has been removed, thus allowing us to directly estimate the effect of BMI on standardized medical expenditures.
To estimate the impact of these factors we used penalized regression on the L_2 norm (ridge regression) to predict total medical expenditures in a two-part model. The first part of the model predicts the probability of having positive medical expenditures, and the second part of the model predicts the level of expenditure given positive expenditure. For the second stage we excluded individuals with no reported expenditure, leaving 137,736 respondents, and log-transformed expenditure. We back-transformed the predicted level of expenditure from log dollars to dollars using Duan's Smearing Estimator (Duan 1983).

Cross-validation was used to select the optimal penalty value (λ) by minimizing the average binomial deviance (logistic regression) and mean-squared prediction error (linear regression) over 10-folds of the dataset. We used the `glmnet` R package (version 2.0-10) to fit the penalized regression models.

Reference: Duan N. Smearing Estimate: A Nonparametric Retransformation Method. J Am Stat Assoc. 1983;78(383):605-610.

3.1 Part I: Probability of Positive Expenditure

We estimated a logistic regression model to predict the probability of positive expenditure $P(Y > 0)$. Here we report the logistic regression coefficients and 95% CIs.

3.1.1 Adults

Variable	Overall (95% CI)	Males (95% CI)	Females (95% CI)
(Intercept)	1.8976 (1.7896 - 2.0078)	1.18 (1.0625 - 1.2996)	1.9372 (1.7775 - 2.1151)
BMI *	0.1638 (0.1377 - 0.189)	0.163 (0.1293 - 0.1955)	0.1675 (0.1302 - 0.2035)
BMI^2 *	0.0119 (0.0047 - 0.0235)	0.0069 (-9e-04 - 0.0274)	0.0151 (0.0017 - 0.0302)
BMI^3 *	-5e-04 (-7e-04 - 2e-04)	-3e-04 (-0.0028 - 3e-04)	1e-04 (-6e-04 - 0.0015)
Year *	-0.0265 (-0.051 - 0.001)	-0.0176 (-0.0487 - 0.0142)	-0.0416 (-0.0785 - -0.0045)
Year^2 *	-0.0174 (-0.0413 - 0.0064)	-0.0158 (-0.0433 - 0.014)	-0.0221 (-0.0552 - 0.0097)
Year^3 *	-0.0117 (-0.0232 - 2e-04)	-0.0143 (-0.0294 - 0.0011)	-0.008 (-0.0247 - 0.0083)
Region: Northeast (Ref)	—	—	—
Region: Midwest	0.2004 (0.1133 - 0.2876)	0.1887 (0.1 - 0.275)	0.2286 (0.0965 - 0.3718)
Region: South	-0.033 (-0.1031 - 0.0497)	-0.0331 (-0.1122 - 0.0477)	-0.0361 (-0.1399 - 0.0811)
Region: West	-0.0075 (-0.0739 - 0.0692)	0.0072 (-0.0611 - 0.0845)	-0.0289 (-0.1322 - 0.0844)
Age *	0.4801 (0.4436 - 0.5126)	0.5499 (0.5028 - 0.5945)	0.3711 (0.3245 - 0.4232)
Age^2 *	0.1346 (0.1085 - 0.1623)	0.1677 (0.1314 - 0.2035)	0.0977 (0.0592 - 0.1351)
Age^3 *	0.0259 (0.0132 - 0.0391)	0.0282 (0.01 - 0.0496)	0.0275 (0.0099 - 0.045)
Male	-0.6743 (-0.7188 - -0.6342)	0 (0 - 0)	0 (0 - 0)
Race: White (Ref)	—	—	—
Race: Black	-0.4572 (-0.5099 - -0.4021)	-0.4459 (-0.5105 - -0.3813)	-0.5018 (-0.5926 - -0.4196)
Race: AIAN	0.3257 (0.0163 - 0.5671)	0.2879 (-0.0423 - 0.5687)	0.3925 (-0.0205 - 0.8032)
Race: Asian/NH/PI	-0.6833 (-0.7683 - -0.5926)	-0.5426 (-0.6349 - -0.4504)	-0.883 (-1.0188 - -0.7445)
Race: Multi	-0.0624 (-0.2111 - 0.0886)	-0.1189 (-0.302 - 0.0825)	0.049 (-0.1708 - 0.3009)
Hispanic	-0.5298 (-0.6063 - -0.4484)	-0.5146 (-0.5996 - -0.4323)	-0.5657 (-0.6736 - -0.4587)
Marital: Married (Ref)	—	—	—
Marital: Widowed	-0.0833 (-0.2126 - 0.0549)	0.0596 (-0.1514 - 0.2857)	-0.0519 (-0.2245 - 0.1091)
Marital: Divorced	0.0734 (0.0039 - 0.1486)	-0.0155 (-0.1175 - 0.0779)	0.2058 (0.1051 - 0.3021)
Marital: Separated	0.0305 (-0.0818 - 0.1458)	-0.0328 (-0.1895 - 0.1199)	0.1041 (-0.0697 - 0.2971)
Marital: Never Married	-0.1545 (-0.2128 - -0.0974)	-0.1681 (-0.2364 - -0.1023)	-0.0984 (-0.177 - -0.0216)
Education: Unknown (Ref)	—	—	—
Education: <=8th grade	-0.2719 (-0.3442 - -0.2019)	-0.3687 (-0.4582 - -0.2743)	-0.1052 (-0.2092 - 0.0034)
Education: HS, no diploma	-0.2609 (-0.3281 - -0.1976)	-0.2786 (-0.3659 - -0.1855)	-0.2116 (-0.2944 - -0.1255)
Education: GED or diploma	-0.1653 (-0.2052 - -0.125)	-0.1457 (-0.1934 - -0.0971)	-0.1729 (-0.2408 - -0.1055)
Education: Some College	0.1067 (0.063 - 0.1517)	0.0874 (0.0338 - 0.1411)	0.139 (0.0644 - 0.2048)
Education: 4-yr College	0.2938 (0.236 - 0.3507)	0.2858 (0.2088 - 0.3571)	0.2987 (0.205 - 0.3916)
Education: Graduate School	0.4543 (0.3912 - 0.53)	0.4664 (0.379 - 0.5587)	0.3982 (0.2858 - 0.5302)
Smoker	-0.04 (-0.0927 - 0.0155)	-0.0583 (-0.1246 - 0.0103)	0.0024 (-0.0927 - 0.0977)
Poverty Level *	0.1168 (0.09 - 0.1425)	0.1222 (0.091 - 0.1539)	0.1261 (0.08 - 0.1711)
Poverty Level^2 *	0.0065 (5e-04 - 0.0129)	0.0027 (-0.0039 - 0.0095)	0.0131 (0.0028 - 0.0246)
Poverty Level^3 *	-8e-04 (-0.002 - 3e-04)	-0.0013 (-0.0028 - 0)	0.001 (-3e-04 - 0.0027)
Insurance: Private	0.2975 (0.2332 - 0.3577)	0.2829 (0.2051 - 0.3571)	0.3491 (0.2609 - 0.4338)
Insurance: TRICARE	0.4377 (0.2868 - 0.6029)	0.4453 (0.2653 - 0.6338)	0.4336 (0.1737 - 0.7418)
Insurance: Medicare	0.6175 (0.5534 - 0.6864)	0.6393 (0.5448 - 0.7366)	0.6025 (0.5149 - 0.6839)
Insurance: Medicaid	0.3983 (0.3381 - 0.4628)	0.46 (0.3756 - 0.5477)	0.3107 (0.2203 - 0.4028)
Insurance: Public A	0.1921 (-0.1069 - 0.4941)	0.3097 (-0.1842 - 0.7842)	0.0607 (-0.2827 - 0.4577)
Insurance: Public B	0.2545 (0.0435 - 0.4893)	0.359 (0.0929 - 0.6479)	0.112 (-0.1828 - 0.4726)
Insurance: None	-0.9179 (-0.9817 - -0.8541)	-0.8706 (-0.9514 - -0.7885)	-0.9748 (-1.0709 - -0.8828)

* indicates that the variable has been standardized (i.e. mean=0, sd=1)

3.1.2 Children

Variable	Overall (95% CI)	Males (95% CI)	Females (95% CI)
(Intercept)	0.8032 (0.4466 - 1.1334)	-0.3089 (-0.8195 - 0.268)	1.9232 (1.529 - 2.378)
BMI *	-0.0174 (-0.0862 - 0.0423)	-0.0216 (-0.1032 - 0.062)	-0.0238 (-0.1114 - 0.0493)
BMIˆ2 *	0.0184 (-0.0158 - 0.0546)	0.021 (-0.025 - 0.0721)	0.0111 (-0.0289 - 0.0557)
BMIˆ3 *	0.0032 (-0.0073 - 0.0205)	0.0057 (-0.0064 - 0.0219)	0.0023 (-0.0084 - 0.0243)
Year *	0.0218 (-0.0503 - 0.0994)	0.0475 (-0.0411 - 0.142)	-0.0043 (-0.0742 - 0.0836)
Yearˆ2 *	-0.0379 (-0.0844 - 0.0065)	-0.0379 (-0.1071 - 0.0273)	-0.037 (-0.0897 - 0.0233)
Yearˆ3 *	-0.0148 (-0.047 - 0.0173)	-0.0451 (-0.0859 - -0.0053)	0.0203 (-0.0152 - 0.0551)
Region: Northeast (Ref)	—	—	—
Region: Midwest	0.1202 (-0.0494 - 0.2926)	0.1863 (-0.0044 - 0.379)	0.0494 (-0.1618 - 0.2597)
Region: South	-0.2401 (-0.3834 - -0.096)	-0.2295 (-0.3816 - -0.0818)	-0.2528 (-0.4308 - -0.0492)
Region: West	-0.1617 (-0.3148 - -0.0148)	-0.0867 (-0.2402 - 0.0723)	-0.2541 (-0.4548 - -0.0508)
Age *	-1.1061 (-1.375 - -0.8675)	-1.6798 (-2.031 - -1.0152)	-0.3423 (-0.5839 - -0.0297)
Ageˆ2 *	0.1214 (0.0893 - 0.1532)	0.2038 (0.1537 - 0.2574)	0.0311 (-0.0058 - 0.0783)
Ageˆ3 *	0.0634 (0.0204 - 0.1086)	0.0856 (-0.0171 - 0.1473)	0.0186 (-0.033 - 0.0621)
Male	-0.2172 (-0.2894 - -0.1382)	0 (0 - 0)	0 (0 - 0)
Race: White (Ref)	—	—	—
Race: Black	-0.5639 (-0.6667 - -0.4538)	-0.5083 (-0.6763 - -0.3542)	-0.6227 (-0.7226 - -0.4479)
Race: AIAN	-0.0489 (-0.6422 - 0.4831)	0.1105 (-0.7147 - 0.6427)	-0.2365 (-0.7799 - 0.6736)
Race: Asian/NH/PI	-0.6907 (-0.8784 - -0.5019)	-0.603 (-0.8746 - -0.3504)	-0.7973 (-0.9953 - -0.4727)
Race: Multi	-0.1521 (-0.3611 - 0.0836)	-0.1002 (-0.3526 - 0.1733)	-0.1893 (-0.4484 - 0.1825)
Hispanic	-0.3927 (-0.5055 - -0.2743)	-0.3573 (-0.4953 - -0.2117)	-0.4262 (-0.5548 - -0.251)
Smoker	-0.2176 (-0.5025 - 0.0844)	-0.3375 (-0.6739 - -6e-04)	0.4367 (-0.1235 - 1.0333)
Poverty Level *	0.4198 (0.3358 - 0.493)	0.4255 (0.3094 - 0.5171)	0.4175 (0.2222 - 0.5233)
Poverty Levelˆ2 *	-0.0229 (-0.0585 - 0.01)	-0.011 (-0.0507 - 0.0332)	-0.041 (-0.0838 - 0.0171)
Poverty Levelˆ3 *	-0.0019 (-0.0082 - 0.0068)	-0.0066 (-0.0172 - 0.0065)	0.0057 (0.0016 - 0.0104)
Insurance: Private	0.1096 (3e-04 - 0.2344)	0.1146 (-0.0105 - 0.2632)	0.1093 (-0.0281 - 0.2898)
Insurance: TRICARE	0.4743 (0.1749 - 0.8865)	0.5969 (0.2186 - 1.1036)	0.3095 (-0.0905 - 0.8695)
Insurance: Medicare	0.2836 (-0.3634 - 1.3874)	0.4385 (-0.2098 - 1.7007)	0.1168 (-1.1497 - 1.6134)
Insurance: Medicaid	0.0496 (-0.0745 - 0.1872)	0.0591 (-0.0984 - 0.2205)	0.0458 (-0.1019 - 0.1933)
Insurance: Public A	0.2058 (-1.0169 - 2.2491)	-0.1606 (-1.9406 - 2.5325)	0.5908 (-0.5171 - 2.3233)
Insurance: Public B	0.0326 (-0.5305 - 1.0028)	0.0643 (-0.7128 - 1.2464)	-0.0948 (-0.7811 - 1.0534)
Insurance: None	-1.286 (-1.4668 - -1.0932)	-1.2711 (-1.505 - -1.0076)	-1.3065 (-1.4947 - -1.0463)

* indicates that the variable has been standardized (i.e. mean=0, sd=1)

3.2 Part II: Level of Expenditure

We estimated a linear regression model to predict the level of log expenditure, given positive expenditure: $E[\log(Y)|Y > 0]$. Here we report the regression coefficients and 95% CIs.

3.2.1 Adults

Variable	Overall (95% CI)	Males (95% CI)	Females (95% CI)
(Intercept)	7.1125 (7.0378 - 7.1914)	6.8257 (6.7433 - 7.0067)	7.1677 (7.071 - 7.2784)
BMI *	0.1503 (0.1321 - 0.1672)	0.1404 (0.1062 - 0.1687)	0.1642 (0.1417 - 0.1826)
BMI^2 *	0.0173 (0.0095 - 0.0348)	0.0222 (0.0052 - 0.0705)	0.012 (0.0043 - 0.0319)
BMI^3 *	-0.001 (-0.0032 - -6e-04)	-0.0013 (-0.0101 - -2e-04)	-8e-04 (-0.0043 - -4e-04)
Year *	0.0225 (-0.0036 - 0.0485)	0.0144 (-0.0186 - 0.0455)	0.0288 (-0.0029 - 0.0597)
Year^2 *	0.0177 (0.0043 - 0.0305)	0.0166 (-0.0013 - 0.0346)	0.0191 (-1e-04 - 0.038)
Year^3 *	-0.0138 (-0.0251 - -0.002)	-0.0168 (-0.0316 - -2e-04)	-0.0114 (-0.0248 - 0.0026)
Region: Northeast (Ref)	—	—	—
Region: Midwest	-0.0217 (-0.0765 - 0.0387)	0.0279 (-0.0309 - 0.0977)	-0.0681 (-0.1351 - 0.0072)
Region: South	-0.0755 (-0.123 - -0.0248)	-0.0598 (-0.124 - -9e-04)	-0.0919 (-0.1557 - -0.0181)
Region: West	-0.0692 (-0.1226 - -0.0151)	-0.0686 (-0.1302 - -0.004)	-0.071 (-0.1365 - 0.0061)
Age *	0.5787 (0.4748 - 0.6072)	0.6315 (0.3927 - 0.6692)	0.5275 (0.4027 - 0.5584)
Age^2 *	-0.0147 (-0.0379 - 0.0154)	-0.0018 (-0.0318 - 0.058)	-0.0262 (-0.0546 - 0.0132)
Age^3 *	-0.0218 (-0.0326 - -5e-04)	-0.0192 (-0.0334 - 0.0232)	-0.0213 (-0.0339 - 0.002)
Male	-0.2271 (-0.2573 - -0.1998)	0 (0 - 0)	0 (0 - 0)
Race: White (Ref)	—	—	—
Race: Black	-0.2837 (-0.3191 - -0.238)	-0.2414 (-0.2884 - -0.1564)	-0.3233 (-0.3789 - -0.249)
Race: AIAN	0.1022 (-0.0365 - 0.2149)	0.0188 (-0.1893 - 0.2162)	0.1633 (-0.0387 - 0.3139)
Race: Asian/NH/PI	-0.4421 (-0.5006 - -0.3836)	-0.3393 (-0.4088 - -0.25)	-0.5232 (-0.5945 - -0.4445)
Race: Multi	0.1131 (0.0043 - 0.2141)	0.2133 (0.0592 - 0.3462)	0.0289 (-0.0996 - 0.1455)
Hispanic	-0.3038 (-0.3454 - -0.2581)	-0.2635 (-0.3159 - -0.2033)	-0.3428 (-0.3937 - -0.2859)
Marital: Married (Ref)	—	—	—
Marital: Widowed	0.0295 (-0.0259 - 0.0797)	-0.0144 (-0.1159 - 0.0797)	0.1398 (0.0713 - 0.2032)
Marital: Divorced	0.1086 (0.0723 - 0.1448)	0.0368 (-0.0211 - 0.1011)	0.1717 (0.1247 - 0.2167)
Marital: Separated	0.1172 (0.0288 - 0.1955)	0.0882 (-0.0503 - 0.2169)	0.1471 (0.0448 - 0.2422)
Marital: Never Married	0.0757 (0.0097 - 0.1151)	0.107 (-0.0537 - 0.157)	0.0566 (-0.0279 - 0.1033)
Education: Unknown (Ref)	—	—	—
Education: <=8th grade	-0.1282 (-0.1879 - -0.0723)	-0.1883 (-0.2795 - -0.0925)	-0.0794 (-0.1475 - -0.016)
Education: HS, no diploma	-0.0761 (-0.1346 - -0.0211)	-0.0355 (-0.1225 - 0.0419)	-0.1016 (-0.1691 - -0.0363)
Education: GED or diploma	-0.0509 (-0.0844 - -0.0148)	-0.0011 (-0.0505 - 0.0411)	-0.0874 (-0.1295 - -0.0447)
Education: Some College	0.063 (0.0241 - 0.0991)	0.0529 (-0.0053 - 0.0977)	0.0732 (0.0307 - 0.1102)
Education: 4-yr College	0.0559 (0.0241 - 0.0868)	0.0359 (-0.0115 - 0.0754)	0.0642 (0.0219 - 0.1039)
Education: Graduate School	0.1439 (0.1049 - 0.1846)	0.0763 (0.0234 - 0.1327)	0.1838 (0.1238 - 0.2338)
Smoker	0.0597 (0.0249 - 0.097)	0.0283 (-0.0242 - 0.0825)	0.0805 (0.0252 - 0.1337)
Poverty Level *	0.0546 (0.0367 - 0.0715)	0.0455 (0.0225 - 0.0725)	0.071 (0.0484 - 0.0903)
Poverty Level^2 *	0.0055 (-2e-04 - 0.011)	0.0117 (0.0037 - 0.0177)	-7e-04 (-0.0082 - 0.0066)
Poverty Level^3 *	-0.0013 (-0.0021 - -5e-04)	-0.0016 (-0.0025 - -2e-04)	-0.0011 (-0.0021 - -2e-04)
Insurance: Private	0.1451 (0.1028 - 0.1808)	0.0977 (0.0143 - 0.1442)	0.183 (0.1233 - 0.228)
Insurance: TRICARE	0.2162 (0.1429 - 0.2796)	0.2062 (0.1014 - 0.2937)	0.201 (0.0823 - 0.2997)
Insurance: Medicare	0.5955 (0.5339 - 0.6396)	0.6466 (0.5065 - 0.7084)	0.5354 (0.4563 - 0.593)
Insurance: Medicaid	0.4378 (0.382 - 0.4836)	0.4933 (0.3592 - 0.5605)	0.4037 (0.3183 - 0.4571)
Insurance: Public A	-0.0156 (-0.2367 - 0.177)	0.0127 (-0.3537 - 0.3487)	-0.0264 (-0.2482 - 0.1804)
Insurance: Public B	0.1535 (0.0366 - 0.2585)	0.1828 (0.0087 - 0.3331)	0.1355 (-0.0233 - 0.2791)
Insurance: None	-0.5659 (-0.6199 - -0.5126)	-0.5472 (-0.6292 - -0.4611)	-0.5866 (-0.6602 - -0.5194)

* indicates that the variable has been standardized (i.e. mean=0, sd=1)

3.2.2 Children

Variable	Overall (95% CI)	Males (95% CI)	Females (95% CI)
(Intercept)	6.4744 (6.112 - 6.9359)	6.106 (5.5433 - 7.0658)	6.7705 (6.3665 - 7.4597)
BMI *	0.0307 (-0.0197 - 0.0759)	0.051 (-0.011 - 0.1009)	0.0171 (-0.049 - 0.0738)
BMI^2 *	0.0455 (0.0188 - 0.0719)	0.0634 (0.0276 - 0.0904)	0.0293 (-0.0096 - 0.0652)
BMI^3 *	-1e-04 (-0.0067 - 0.0109)	-0.0024 (-0.0095 - 0.0083)	0.0024 (-0.0074 - 0.0185)
Year *	0.0374 (-0.0156 - 0.0938)	-0.0275 (-0.0955 - 0.0506)	0.0996 (0.0277 - 0.1614)
Year^2 *	-0.0213 (-0.0463 - 0.0051)	-0.0171 (-0.0494 - 0.0186)	-0.0262 (-0.0604 - 0.0079)
Year^3 *	-0.0133 (-0.0409 - 0.0136)	0.0108 (-0.0274 - 0.0462)	-0.0367 (-0.067 - 0.0013)
Region: Northeast (Ref)	—	—	—
Region: Midwest	-0.0413 (-0.1297 - 0.046)	-0.051 (-0.1557 - 0.0653)	-0.0264 (-0.123 - 0.0916)
Region: South	-0.1548 (-0.2329 - -0.0769)	-0.1327 (-0.2271 - -0.0354)	-0.1783 (-0.2729 - -0.0668)
Region: West	-0.1433 (-0.2215 - -0.0565)	-0.1146 (-0.2095 - -0.0065)	-0.1756 (-0.2733 - -0.0653)
Age *	-0.7068 (-1.0664 - -0.1199)	-0.8912 (-1.4958 - 0.0924)	-0.5763 (-0.9765 - 0.1691)
Age^2 *	-0.1231 (-0.1429 - -0.0985)	-0.1086 (-0.1314 - -0.0512)	-0.1372 (-0.1627 - -0.0919)
Age^3 *	0.2041 (0.1098 - 0.2674)	0.2134 (0.0446 - 0.3178)	0.2017 (0.0708 - 0.274)
Male	-0.0099 (-0.0549 - 0.0371)	0 (0 - 0)	0 (0 - 0)
Race: White (Ref)	—	—	—
Race: Black	-0.3725 (-0.4375 - -0.3103)	-0.3283 (-0.4115 - -0.2282)	-0.4109 (-0.504 - -0.3058)
Race: AIAN	0.2133 (-0.1274 - 0.445)	0.3579 (-0.1238 - 0.6016)	0.0283 (-0.2646 - 0.299)
Race: Asian/NH/PI	-0.4362 (-0.5318 - -0.3459)	-0.4233 (-0.5542 - -0.2753)	-0.4381 (-0.5501 - -0.3019)
Race: Multi	9e-04 (-0.1256 - 0.1193)	0.0257 (-0.1471 - 0.1937)	-0.0223 (-0.1616 - 0.1165)
Hispanic	-0.3243 (-0.3805 - -0.2579)	-0.3354 (-0.403 - -0.2455)	-0.3062 (-0.3804 - -0.2173)
Smoker	-0.2691 (-0.5093 - -0.0221)	-0.3653 (-0.6689 - -0.0352)	-0.0732 (-0.4991 - 0.3119)
Poverty Level *	0.2273 (0.1782 - 0.2755)	0.1917 (0.1128 - 0.2511)	0.2608 (0.1695 - 0.3226)
Poverty Level^2 *	-0.0091 (-0.0367 - 0.0161)	0.0011 (-0.0354 - 0.0324)	-0.0173 (-0.0508 - 0.0136)
Poverty Level^3 *	-0.0035 (-0.009 - 0.0024)	-0.0041 (-0.0101 - 0.0019)	-0.0032 (-0.0102 - 0.0046)
Insurance: Private	0.2439 (0.1419 - 0.3466)	0.3137 (0.1439 - 0.4453)	0.1842 (0.0567 - 0.31)
Insurance: TRICARE	0.2723 (0.083 - 0.4394)	0.28 (0.0057 - 0.4878)	0.2541 (0.0044 - 0.4727)
Insurance: Medicare	0.3447 (-0.0569 - 1.0161)	0.3324 (-0.3281 - 0.9871)	0.3513 (-0.1999 - 1.5048)
Insurance: Medicaid	0.0919 (-0.0193 - 0.1993)	0.1837 (-0.0182 - 0.3219)	0.0027 (-0.1321 - 0.128)
Insurance: Public A	0.3689 (-0.1977 - 0.7874)	-0.344 (-1.1074 - 0.2911)	0.7767 (-0.0041 - 1.1786)
Insurance: Public B	0.5777 (0.2468 - 0.8674)	0.3619 (-0.1628 - 0.8973)	0.7652 (0.2611 - 1.1033)
Insurance: None	-0.4756 (-0.6323 - -0.3318)	-0.4187 (-0.628 - -0.2102)	-0.5176 (-0.7154 - -0.3279)

* indicates that the variable has been standardized (i.e. mean=0, sd=1)

3.3 Full Model Standardization

For the first-part of the model (logistic regression), we adjusted the probability of positive expenditure for each individual to be representative of a standardized individual.

For the second part of the model (linear regression), we adjusted respondents' log expenditures according to their distance from the synthetic, average person. (Higher order terms (square/cubic) were calculated from the mean base value. For example, the mean value for the age-squared term was calculated as $(a\bar{g}e)^2$ instead of $\overline{age^2}$).

Each person's expenditures was then adjusted by their distance from the mean of each variable using the relevant coefficients. We adjusted for all variables except BMI (and age when fitting the bivariate prediction model, see Appendix 4), thus controlling for other salient factors.

Here we plot the distribution of standardized vs crude expenditures by adjusted BMI.

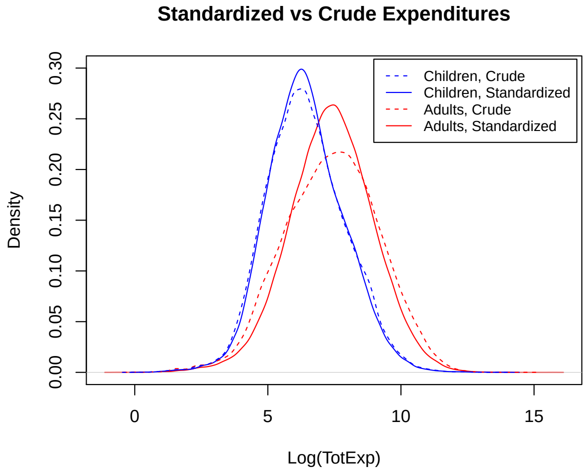

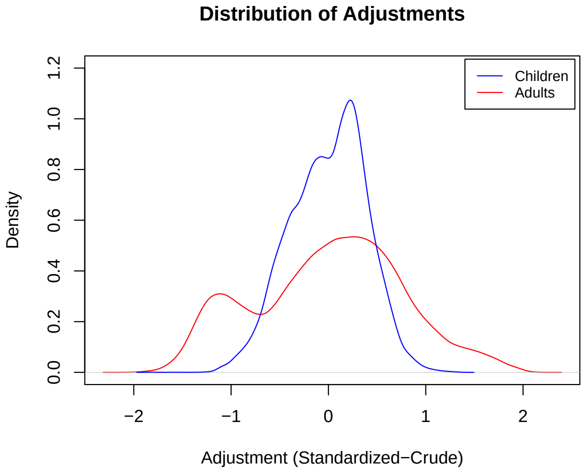

4 BMI-related Expenditure Prediction

4.1 Continuous

After standardizing the probability and level of expenditures we predicted total expenditure as a function of BMI alone (1-dimensional) using cubic smoothing splines, and as a function of both BMI and Age (2-dimensional) using generalized additive models (GAMs). We fit separate models using self-reported and adjusted BMI to explore the impact of self-report bias.

While data from respondents with BMI>80 (n=21) were included when fitting models, we did not predict expenditures for BMI greater than 80 due to sparse data in this extreme range.

Univariate - Smooth Spline

Cubic spline regression is a flexible modeling approach in which piecewise cubic polynomials are fit to data. Rather than trying to fit a single functional form (e.g. n-order polynomial) to all datapoints, the problem is split into smaller pieces. Specifically, the data is fitted to a set of cubic spline basis functions defined by a set of 'knots' on the domain.

Using cubic polynomials allows the flexibility to fit to local data while ensuring that the global fit is smooth. However, it can be sensitive to the number and location of knots chosen.

Using smoothing splines avoids the problem of knot selection by using a maximal set of knots (i.e. using each unique datapoint on the domain), and regularizing the fit (i.e. shrinking the coefficients of the estimated basis expansion function) via a smoothing parameter. This is accomplished by minimizing the penalized residual sum of squares (RSS):

$$RSS(f, \lambda) = \sum_{i=1}^{N} (y_i - f(x_i))^2 + \lambda \int (f''(t))^2 \, dt$$

where $f(x)$ in this case is a cubic polynomial and λ is a smoothing parameter. The first term measures closeness to the data, while the second term penalizes curvature in the function, with λ controlling the trade-off between the two terms. We used cross-validation to determine λ.

We used the `smooth.spline` command in the `stats` R package (package version 3.3.1).

Bivariate - GAMs

GAMs have the advantage that relationships between individual predictors and the dependent variable (expenditures) follow smooth patterns (linear or nonlinear) that can be estimated simultaneously, with the final function calculated by simply adding up the terms.

We used the `gam` command in the `mgcv` R package (version 1.8-12) which fits a GAM using a quadratically penalized likelihood approach while also estimating the degree of smoothness via cross-validation. We did not standardize expenditures for age when fitting the bivariate model as this would have removed the variation in this dimension.

We fit a GAM with tensor product smooths which have one penalty per marginal basis (i.e. different penalties for age and BMI), allowing the degree of smoothness to vary by dimension. This allowed us to fit a smooth surface of predicted expenditures while estimating the interaction of BMI and age on medical expenditures in a non-parametric way.

4.2 Categorical

With the predicted expenditures by continuous BMI we then estimated categorical costs based on individuals in each BMI category. We estimated categorical expenditures by binary obesity status and by 5 BMI categories. We defined the BMI thresholds for each category based on the CDC definitions.

BMI Category	Children (6-19)*	Adults (20+)
Non-Obesity	$\text{BMI} < 95\%_{ile}$	$\text{BMI} < 30$
Obesity	$\text{BMI} \geq 95\%_{ile}$	$\text{BMI} \geq 30$
BMI Category		
Underweight	$\text{BMI} < 5\%_{ile}$	$\text{BMI} < 18.5$
Normal weight	$5\%_{ile} \leq \text{BMI} < 85\%_{ile}$	$18.5 \leq \text{BMI} < 25$
Overweight	$85\%_{ile} \leq \text{BMI} < 95\%_{ile}$	$25 \leq \text{BMI} < 30$
Moderate Obesity	$95\%_{ile} \leq \text{BMI} < 120\% * 95\%_{ile}$	$30 \leq \text{BMI} < 35$
Severe Obesity	$\text{BMI} \geq 120\% * 95\%_{ile}$	$\text{BMI} \geq 35$

* $\%_{ile}$: percentile

References:

Defining Childhood Obesity. Atlanta (GA): Centers for Disease Control and Prevention. Available from: https://www.cdc.gov/obesity/childhood/defining.html.

Defining Adult Overweight and Obesity. Atlanta (GA): Centers for Disease Control and Prevention. Available from: https://www.cdc.gov/obesity/adult/defining.html.

5 Model Uncertainty

To estimate 95% CIs we bootstrapped the MEPS dataset 1,000 times and re-estimated all models (i.e. two-part expenditure standardization model and univariate/bivariate GAMs), taking the 2.5 and 97.5 percentiles of the bootstrapped results. In order to take into account the complex survey design of MEPS we created bootstrap replicate weights in each iteration by sampling PSUs within each stratum. Specifically, given a stratum that contains N PSUs we sampled $N - 1$ PSUs with replacement from the stratum and calculated the replicate weights for each individual i as:

$$w_i f \frac{N}{N-1}$$

where w_i is the original weight and f is the number of times the PSU to which the individual belongs was sampled.

Reference: Tihomir Asparouhov and Bengt Muthen. Resampling Methods in Mplus for Complex Survey Data. 2010. https://www.statmodel.com/download/Resampling_Methods5.pdf

6 Comparisons to Previous Estimates

Here we compare our estimates to previously published estimates. Our results benefit from several methodological advantages. For example, our novel approach to adjust for self-report bias allows us to maintain the shape of the entire BMI distribution. Additionally, our continuous models use data from all points of the distribution. We are thus able to estimate the cost impact of BMI changes at any point of the distribution, rather than only at specific category thresholds. In addition, our use of log expenditure and penalized regression allowed us to fit models that use all available data without having to exclude extreme values, as has been done in previous analyses.

6.1 Finkelstein 2008

We obtained obesity-related cost estimates from Finkelstein 2008 (based on MEPS 2001-2003) and inflated them to $US 2019 using the medical CPI. Using these age-specific estimates we predicted the cost for each person in our MEPS dataset based on obesity status.

Note: It is not clear whether the "Normal" category in this analysis refers to non-obesity (BMI < 30), or normal weight ($18.5 \leq$ BMI < 25). Given that only 2 categories of costs are presented we assumed that obesity was considered as a binary category and estimated "Normal" expenditures for respondents with BMI < 30.

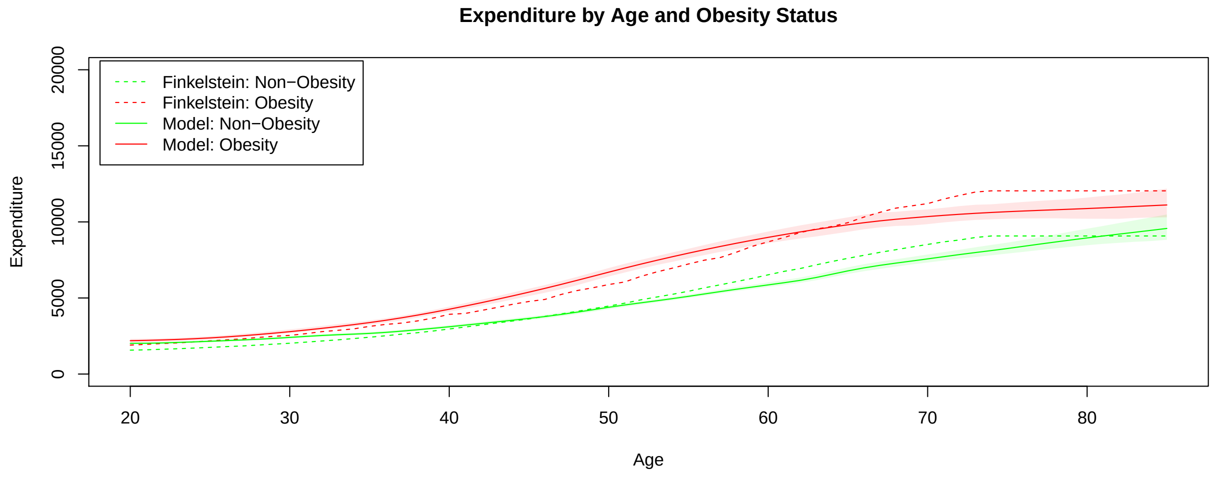

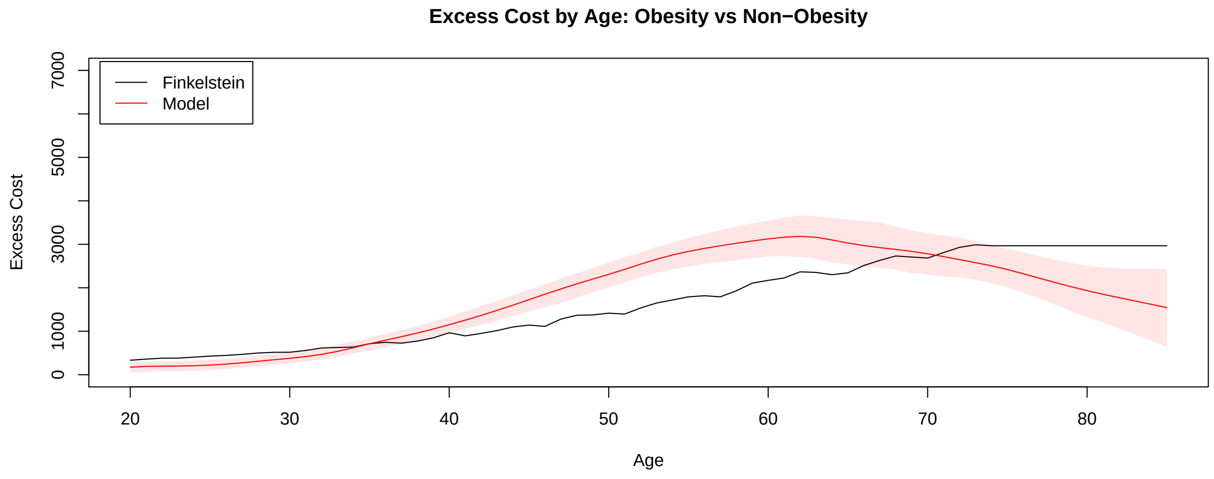

Reference:

Finkelstein EA, Brown DS. Why does the private sector underinvest in obesity prevention and treatment? N C Med J. 2006;67:310-312.
Reproduced in: Finkelstein EA, Trogdon JG. Public health interventions for addressing childhood overweight: Analysis of the business case. American Journal of Public Health. 2008;98(3):411-5.

6.2 Wang 2015

We obtained cost estimates for adults by obesity category from Wang 2015 (based on MEPS 2007-12) and inflated them to $US 2019 using the medical CPI. Using these estimates by sex and age group (18-44, 45-64, 65+) we predicted the cost for each person in our MEPS dataset based on their obesity category (moderate obesity, severe obesity). We then predicted the costs for each person in our dataset using both our univariate (age-standardized) and bivariate (continuous age) models. We see that our predicted costs are higher since we take into account the non-linear interaction of BMI and age. We did not exclude respondents with expenditures over $100,000 as was done in the Wang analysis, which may also explain our higher estimated costs for severe obesity.

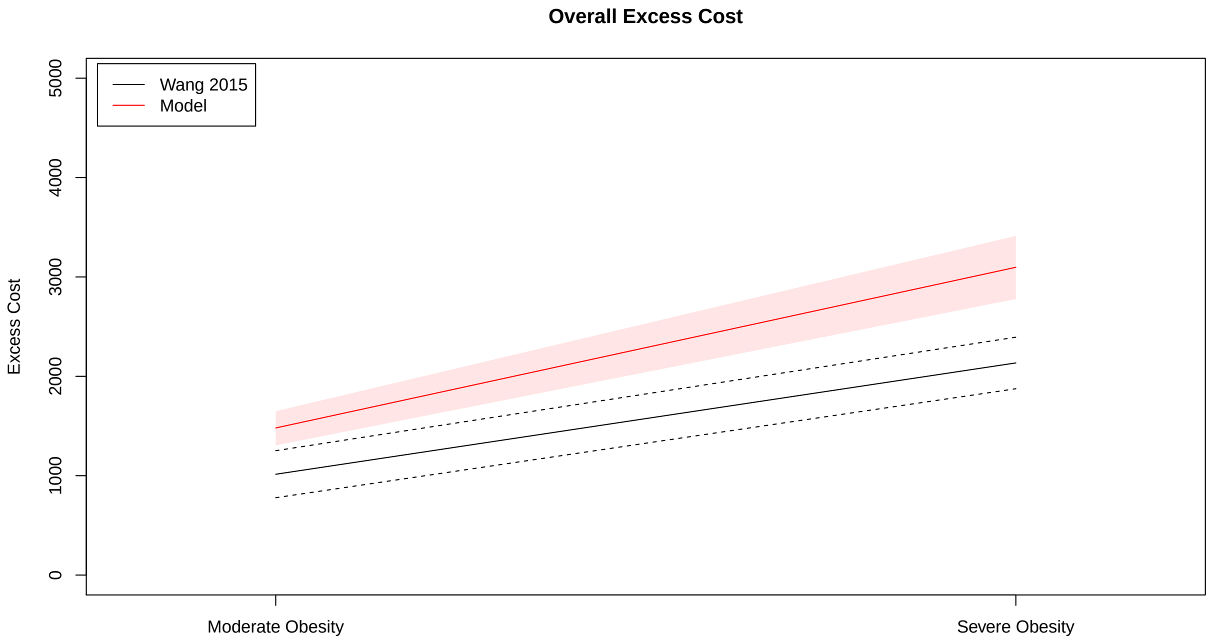

Reference:
Wang CY, Pamplin J, Long MW, Ward ZJ, Gortmaker SL, Andreyeva T. Severe Obesity In Adults Cost State Medicaid Programs Nearly $8 Billion In 2013. Health Aff (Milwood). 2015;34(11):1923-31.

6.3 Cawley and Meyerhoefer 2012

The results from the instrumental variable (IV) analysis by Cawley and Meyerhoefer (based on restricted use data from MEPS 2000-05) indicate that obesity raises annual medical costs (in 2005 $US) by $2741 overall, with increases of $1152 for men and $3613 for women. However, it is not clear how generalizable these results are to the entire adult population. The authors note that due to the “instrument we use in our IV models, we are forced to limit our sample to adults with a biological child between the ages of 11 and 20 years.” As a comparison, we restricted our sample to adults with children using the parent identifiers available in the public MEPS datasets. While these publically-available identifiers do not distinguish between biological, adopted, or step children, they should still provide a comparable sample of adults as used in the IV analysis. Following MEPS guidelines, we ensured that mothers are at least 12 years older than the child and no more than 55 years older, and that fathers are at least 12 years older than the child.

Here we compare our sample characteristics of adults with children to the sample used in the IV analysis. (All means are weighted.)

	IV Analysis	Ward Analysis
Years	2000-2005	2011-2016
Males		
Sample size	9,852	13,945
BMI - self (SD)	28.17 (4.88)	29.11 (5.79)
White	0.72	0.62
Hispanic	0.14	0.20
Black	0.09	0.10
Other race	0.05	0.08
Age 20-34	0.06	0.08
Age 35-44	0.43	0.33
Age 45-54	0.43	0.42
Age 55-64	0.09	0.15
Females		
Sample size	13,837	19,249
BMI - self (SD)	27.37 (6.42)	28.43 (6.95)
White	0.66	0.58
Hispanic	0.15	0.20
Black	0.14	0.13
Other race	0.06	0.08
Age 20-34	0.11	0.11
Age 35-44	0.52	0.41
Age 45-54	0.34	0.38
Age 55-64	0.03	0.08

We see below that the age distribution of adults with children ages 11-20 is quite different from the general adult population, raising questions about the generalizability of the IV estimates.

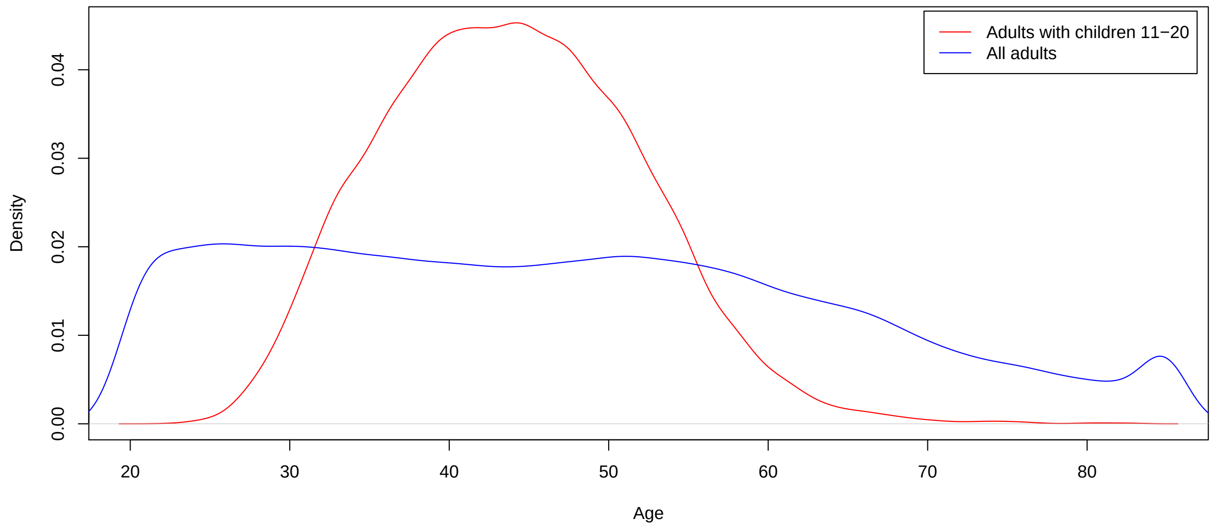

As a comparison of obesity-related expenditures, we inflated the IV estimates to $2019 using the PCE-Health index and applied them to our sample of parents based on obesity status.

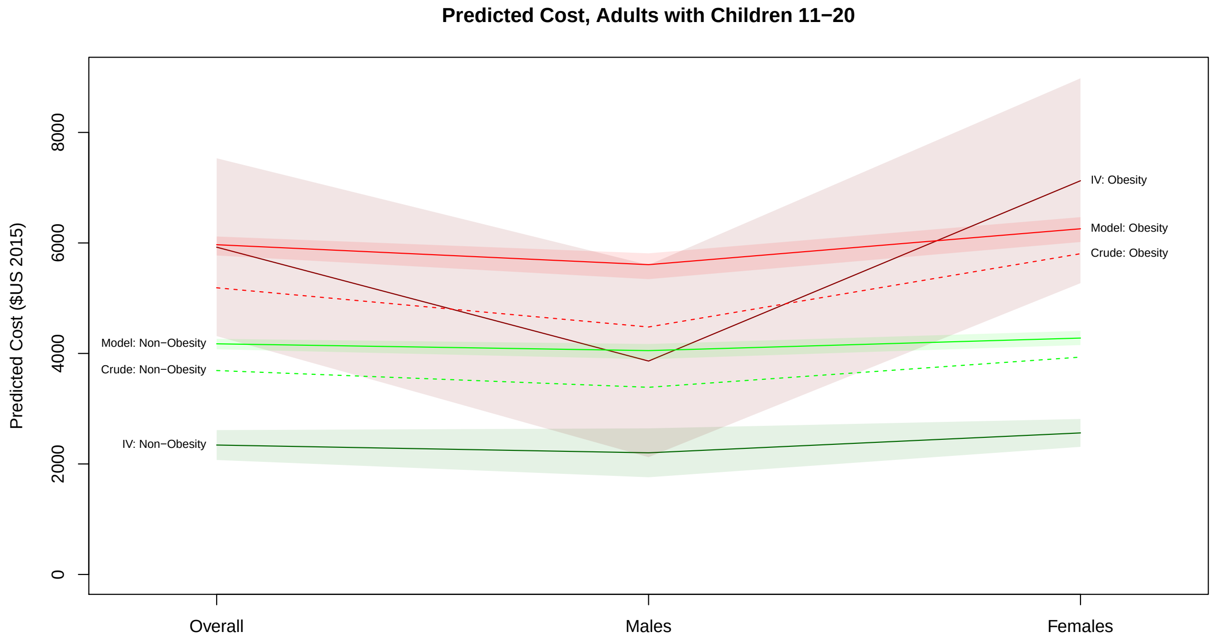

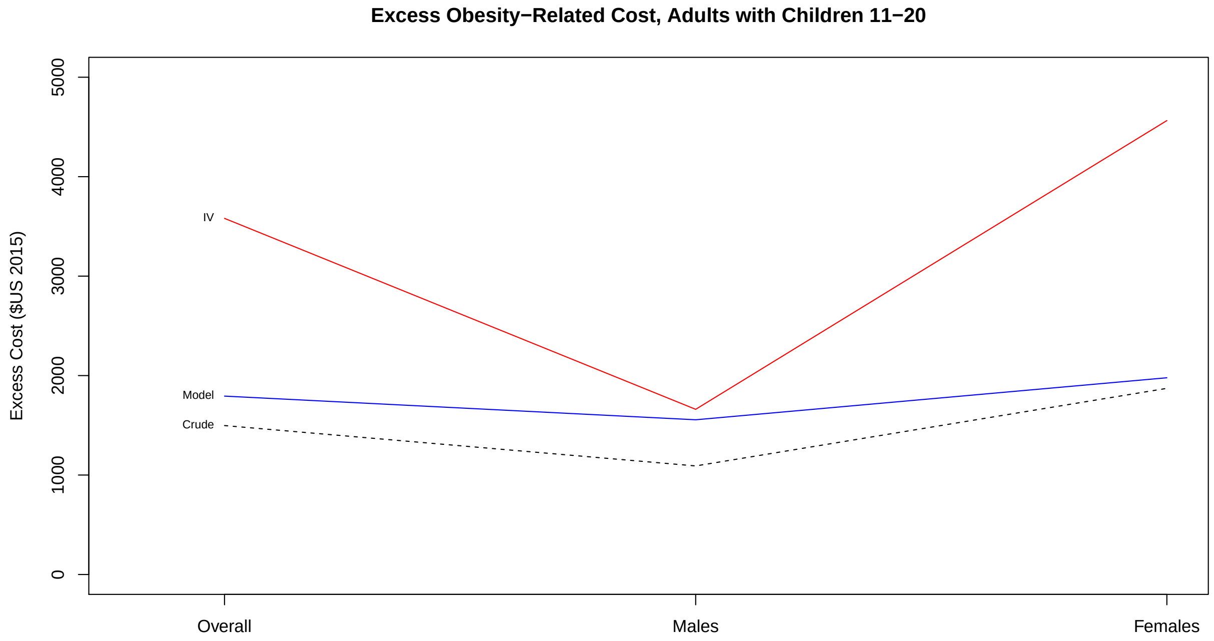

We see that our predicted costs for adults with obesity match the IV results, but that the baseline (non-obesity) costs from the IV analysis are much lower than our predictions. It is this difference which drives the high excess obesity-related costs reported in the paper. However, it is not clear why the IV estimates of non-obesity costs are so much lower than our estimates, and are indeed substantially lower than the crude estimates of non-obesity expenditures in MEPS. In contrast, our estimates result in similar adjustments to the crude MEPS estimates of obesity and non-obesity expenditures, with consistent findings of excess costs for both males and females.

In addition to the lack of generalizability, one concern with the IV analysis may be the validity of the instrument used. The authors note that validity would be threatened if both the respondent and the biological relative are affected by a common household environment that is also directly correlated with the respondent's medical expenditures. Thus shared environmental variables such as sugar sweetened beverage consumption (which is causally linked to obesity and also to diabetes independently of obesity) would threaten the validity of the instrument.

Reference:
Cawley J, Meyerhoefer C. The medical care costs of obesity: An instrumental variables approach. J Health Econ. 2012;31:219-230.
