# Supplementary material for: Association of body mass index with health care expenditures in the United States by age and sex
Source: PLoS One. 2021 Mar 24;16(3):e0247307. doi: 10.1371/journal.pone.0247307 (PMC7990296; doi:10.1371/journal.pone.0247307)
Supplement: Attachment — Submitted filename: Response to reviewers.docx [file pone.0247307.s002.docx]

**We thank the reviewers for their comments. We have provided responses to each comment below (in bold), and have highlighted revisions to the manuscript in blue.**

**Additional Editor Comments:**

1. Please ensure that your manuscript meets PLOS ONE's style requirements, including those for file naming. The PLOS ONE style templates can be found at

<https://journals.plos.org/plosone/s/file?id=wjVg/PLOSOne_formatting_sample_main_body.pdf> and

<https://journals.plos.org/plosone/s/file?id=ba62/PLOSOne_formatting_sample_title_authors_affiliations.pdf>

**We have revised the manuscript formatting and file naming to meet the style requirements.**

2. Please provide additional details regarding participant consent. In the ethics statement in the Methods and online submission information, please ensure that you have specified (1) whether consent was informed and (2) what type you obtained (for instance, written or verbal, and if verbal, how it was documented and witnessed). If your study included minors, state whether you obtained consent from parents or guardians. If the need for consent was waived by the ethics committee, please include this information.

If you are reporting a retrospective study of medical records or archived samples, please ensure that you have discussed whether all data were fully anonymized before you accessed them and/or whether the IRB or ethics committee waived the requirement for informed consent. If patients provided informed written consent to have data from their medical records used in research, please include this information.
Once you have amended this/these statement(s) in the Methods section of the manuscript, please add the same text to the “Ethics Statement” field of the submission form (via “Edit Submission”).

For additional information about PLOS ONE ethical requirements for human subjects research, please refer to <http://journals.plos.org/plosone/s/submission-guidelines#loc-human-subjects-research>.

**We used public use samples of de-identified datasets which are freely available online. We have amended the Methods section of the manuscript to clarify this point.**

**Line 24: “We used publicly available, de-identified data from the Medical Expenditure Panel Survey (MEPS) 2011-2016.”**

3. You have not indicated whether ethical approval was waived or necessary for your study. We understand that the framework for ethical oversight requirements for studies of this type may differ depending on the setting and we would appreciate some further clarification regarding your research. Could you please provide further details on ethical oversight of your study. Please clarify whether or why your study is exempt from the need for approval.

**This work does not involve human subjects, and is thus IRB exempt. We used public use samples of de-identified datasets which are freely available online.**

4. In your Data Availability statement, you have not specified where the minimal data set underlying the results described in your manuscript can be found. PLOS defines a study's minimal data set as the underlying data used to reach the conclusions drawn in the manuscript and any additional data required to replicate the reported study findings in their entirety. All PLOS journals require that the minimal data set be made fully available. For more information about our data policy, please see <http://journals.plos.org/plosone/s/data-availability>.

Upon re-submitting your revised manuscript, please upload your study’s minimal underlying data set as either Supporting Information files or to a stable, public repository and include the relevant URLs, DOIs, or accession numbers within your revised cover letter. For a list of acceptable repositories, please see <http://journals.plos.org/plosone/s/data-availability#loc-recommended-repositories>. Any potentially identifying patient information must be fully anonymized.

Important: If there are ethical or legal restrictions to sharing your data publicly, please explain these restrictions in detail. Please see our guidelines for more information on what we consider unacceptable restrictions to publicly sharing data: <http://journals.plos.org/plosone/s/data-availability#loc-unacceptable-data-access-restrictions>. Note that it is not acceptable for the authors to be the sole named individuals responsible for ensuring data access.

We will update your Data Availability statement to reflect the information you provide in your cover letter.

**We have revised the cover letter to provide this information. We have also added our estimates of age/sex/BMI-specific medical expenditures to a data repository, which will be made publicly available if the analysis is accepted for publication.**

**Lines 132-133:**

**“BMI-specific medical costs by age and sex are available in a public repository (https://doi.org/10.7910/DVN/872OW1).”**

**Reviewers' comments:**

Reviewer #1:

This is a very carefully constructed analysis of the cost of continuously, in addition to categorically, measured BMI considering both age and sex with which I can find no fault in method.

**Thank you for your supportive comments.**

I have two suggestions for improvement in the paper. The first is to explain why you undertake all the corrections for self-report bias relative to NHANES rather than simply using NHANES. It seems like NHANES may not be sufficiently representative, but it is unclear as they cover the same time period.

**Thank you for this clarifying question. NHANES is generally considered the gold-standard for nationally-representative, measured BMI. However, NHANES does not have information on medical costs – the outcome of interest in this analysis. Although MEPS does have individual-level data on both medical costs and BMI, the BMI data are self-reported, which is well-known to cause bias – generally underestimating BMI for adults, and often overestimating it for young children. Because NHANES and MEPS are both designed to be nationally-representative samples, we can use information on the BMI distribution from NHANES to adjust MEPS data for self-reporting bias, using the corrected individual-level BMI to more accurately estimate the relationship between BMI and medical expenditures.**

The second in the development of the conclusions. The paper set out to “To address key gaps in the literature, in this paper we estimate continuous BMI-specific medical costs by age and sex, and provide updated estimates of the medical costs attributable to obesity using recent data.” Please link back to this in the conclusions—note the higher costs of females, the comparatively low cost of children, and the high costs of 60-70 year olds. Linking back the billions of dollars in excess costs as a motivation (including opportunity cost) as an immediate call to policy action could be emphasized.

**Thank you for this suggestion. We have highlighted these findings in the conclusions.**

**Lines 194-203:**

**“We found that health care expenditures are higher for people with excess weight across a wide range of ages and BMI levels, with especially high costs for people with severe obesity. Obesity-related medical costs are higher for adult females, and increase with age for all adults, with the highest estimated costs occurring for 60-70 year olds. Although childhood obesity contributes a small proportion of total obesity-related medical costs, because excess weight in childhood is a strong predictor of adult obesity, policies to prevent excess weight gain at all ages are needed to mitigate the health and economic impact of the obesity epidemic, which accounts for over $170 billion in excess medical costs per year in the United States. These findings highlight the importance of promoting healthy weight across the entire BMI distribution, and provide policy makers and practitioners with more accurate estimates of the health care cost impact of excess weight by age, sex, and continuous BMI.”**

Reviewer #2:

I appreciate this very relevant and important topic. The data and information as presented for the obesity-related costs by BMI units as well as by BMI percentile categories is very clear. There is limited data out there for pediatrics so greatly appreciate the inclusion of pediatrics from ages 6-19 as well as your ability to control for all the many variables listed and the use of separate models for children vs adults.

**Thank you for your supportive comments. We agree that data for children are often not available, and felt it was important to include them in this analysis**

I did have a question about the use of z-scores in children vs the use of actual BMI values like in the adults as the use of z- scores in the literature has become somewhat controversial.

**Thank you for the opportunity to clarify this point. We did use the actual BMI values when estimating the models, but use the corresponding z-scores to display the predicted expenditures in the figure. We have clarified this in the manuscript.**

**Lines 68-72:**

**“We predicted adult expenditures for BMI values between 10 and 80. For children we predicted expenditures for BMI z-scores between -3 and +3. Although we fitted our model using BMI for both children and adults, we present the univariate predicted expenditures for children in terms of BMI z-score for visual ease, as the overweight and obesity thresholds are defined by BMI z-score for children.”**

You may want to consider reviewing this article below from 2017 that is similar in nature (but nto the same) to your study.
The Additional Costs and Health Effects of a Patient Having Overweight or Obesity: A Computational Model Saeideh Fallah-Fini1,2, Atif Adam1, Lawrence J. Cheskin1, Sarah M. Bartsch1, and Bruce Y. Lee Obesity (2017) 25, 1809-1815. doi:10.1002/oby.21965

**Thank you for this suggestion. The referenced paper estimates incremental costs over an adult’s lifetime using a Markov model, with the direct medical costs of overweight/obesity vs normal weight one of the inputs to the model, based on data from MEPS. However it is not clear that the authors adjusted for self-report bias or potential confounding when analyzing the MEPS data. Our current analysis aims to provide such cost estimates which could be used in simulation models such as this.**
